# Keystone Flap for Closure of Skin Cancer Defects on the Upper Extremity

**DOI:** 10.1177/22925503221094106

**Published:** 2022-04-21

**Authors:** Travis Gordon, Andrew P. Golin, Alexander Anzarut

**Affiliations:** 1 Division of Plastic Surgery, 8166 The University of British Columbia, Vancouver, British Columbia, Canada; 2 Faculty of Medicine, 8166 The University of British Columbia, Vancouver, British Columbia, Canada; 3 Department of Surgery, The University of British Columbia, Vancouver, British Columbia, Canada

**Keywords:** skin neoplasms, surgical flaps, upper extremity, wound closure techniques, reconstructive, Mots-clés, Néoplasies cutanées, volets chirurgicaux, membre supérieur, techniques de fermeture des plaies, reconstruction

## Abstract

**Background:** We sought to examine the efficacy of the Keystone Design Perforator Island Flap (KDPIF) for the reconstruction of skin cancer excision defects isolated to the upper extremity. In particular, to examine the size of defects repaired and the complications associated with the keystone flap procedure isolated to the upper extremity. **Methods:** This is a retrospective chart review including all patients older than 18 years of age who received a KDPIF procedure between February 2013 and February 2019 for the oncologic reconstruction of skin cancer defects isolated to the upper extremities by a single surgeon. All procedures were done according to the original description by Behan. **Results:** A total of 32 patients, 18 (56%) male and 14 (44%) female, received 35 keystone flaps between February 2013 and February 2019. The mean age of the males and females was 70.5 and 79.7 years of age, respectively. Thirty-five lesions suspicious for cancer were excised and 14 (40%) basal cell carcinoma (BCC), 11 (31%) squamous cell carcinoma (SCC), 9 (26%) melanoma, and 1 (3%) actinic keratoses diagnoses were histopathologically determined. Skin defect excisions varied from 3.53 cm^2^ to 31.42 cm^2^. No intraoperative or postoperative complications occurred. **Conclusions:** The keystone flap is a successful versatile flap procedure with a low or absent complication rate for the reconstruction of skin cancer excision defects of various locations (eg arm, hand, elbow, forearm, shoulder, and wrist), cancer pathologies, and sizes on the upper extremity. When needed, a Doppler may successfully identify adequate perforating blood vessels for the relatively larger flaps.

## Résumé

**Contexte:** Nous avons cherché à connaître l’efficacité du lambeau en clé de voûte/îlot appelé « keystone design perforator island flap » (KDPIF) pour la reconstruction de la peau après excision de cancers isolés du membre supérieur. Nous avons plus particulièrement examiné la dimension des tissus manquants et réparés, ainsi que les complications associées à la procédure KDPIF isolée au niveau du membre supérieur. **Méthodes:** Il s’agit d’une étude rétrospective de dossiers incluant tous les patients âgés de plus de 18 ans ayant bénéficié d’une procédure KDPIF entre février 2013 et février 2019 pour reconstruction oncologique de manques de substance isolés après excision de cancers de la peau du membre supérieur par un seul chirurgien. Toutes les procédures ont été exécutées selon la description originale de Behan. **Résultats:** Un total de trente-deux patients (18 hommes [56%] et 14 femmes [44%]) ont bénéficié de trente-cinq volets en clé de voûte entre février 2013 et février 2019. L’âge moyen des patients masculins était de 70.5 ans et celui des patientes féminines était de 79.7 ans. Trente-cinq lésions suspectes de cancer ont été excisées et les diagnostics ont été confirmés par l’histopathologie : 14 (40%) carcinomes basocellulaires, 11 (31%) carcinomes spinocellulaires (à cellules squameuses), 9 (26%) mélanomes et 1 (3%) kératose actinique. La surface de peau manquante due à l’excision était comprise entre 3.53 cm^2^ et 31.42 cm^2^. Aucune complication peropératoire ou postopératoire n’est survenue. **Conclusions:** Le volet en clé de voûte KDPIF est une procédure versatile efficace ayant un taux de complication faible ou nul pour la reconstruction pour manque de peau après excision de cancer cutané à divers emplacements (bras, main, coude, avant-bras, épaule, poignet), des pathologies cancéreuses et des tailles variables sur le membre supérieur. Quand cela est nécessaire, un examen Doppler peut identifier avec succès les vaisseaux sanguins perforants pour les volets relativement plus grands.

## Introduction

With an ageing population, the occurrence of skin cancers is increasing.^1^ After surgical removal of a skin cancer, the wound defect must be closed. Options for closure include direct closure, skin grafts, local flaps, and free flaps. At the extremities, direct closure and local flap closure are frequently not possible due to high skin tension. Therefore, the mainstay for closure of skin excisions has been skin grafting, despite its donor site morbidity,^2^ relatively poor aesthetics^2^ and extended healing time.^3^

Ideally, coverage of skin defects occurs by transposition of locoregional tissue of like-to-like composition, contour, and quality.^4^ The Keystone Design Perforator Island Flap (KDPIF) was originally described by Behan in 2003, and has been an efficacious local flap technique for cancer and traumatic injury reconstruction.^5^ The keystone flap is an islanded flap and is designed as two opposing V-Y advancements flaps, which are supplied by underlying fasciocutaneous and musculocutaneous perforating blood vessels^5,6^ that are preserved during careful intraoperative blunt dissection. It is because of this islanding and reduction of tissue tension that the keystone flap may allow for local flap coverage in situations where flaps were not previously possible.^2^ This has the potential to reduce the number of skin grafts being performed. Advantages of using local flaps include decreased donor site morbidity, improved aesthetics,^7^ and faster healing time.^3^

Since the keystone flap was first described, it has been extensively studied for its utility in the reconstruction in head and neck cancer reconstruction,^8,9^ truncal defect reconstruction,^10^ and limb defect reconstruction.^11–14^ The keystone flap has also been compared directly with skin grafts for skin cancer and traumatic injury reconstruction, and results suggest the keystone Flap is superior to skin grafts in terms of aesthetic outcomes, shorter healing times, and return to weight-bearing status, whereas the keystone flap is equivalent in terms of complication rates, and length of hospital stays compared to skin grafts.^3^ Other advantages of this procedure include its ease of use, reproducibility, versatility, ability to perform under local anaesthetic, low complication rates, and superior aesthetic outcomes.^5,9,11^

Despite the extensive literature studying the keystone flap and its utility as a reconstructive option for skin cancer and traumatic defects, most of these studies have focused on the head and neck, trunk, and lower extremity. To our knowledge, there have been few studies that have included data on the reconstruction of defects located on the upper extremity. In addition, we believe this study includes the greatest number of keystone flaps performed by a single surgeon isolated to the upper extremity. Consequently, the purpose of this paper was to determine the efficacy of the keystone flap for the reconstruction of skin cancer defects isolated to the upper extremity.

## Materials and Methods

This is a retrospective chart review including all patients older than 18 years of age who received a KDPIF procedure at one institution in British Columbia, Canada, between February 2013 and February 2019 for the oncologic reconstruction of skin cancer defects isolated to the upper extremities. All procedures were done according to the original description by Behan and performed by the principal investigator (PI).^5^ If needed, an ultrasound Doppler probe was used preoperatively to identify adequate perforating blood vessels for the relatively larger keystone flaps. Patient demographics, defect location and size (mean cm^2^; range), skin cancer pathology, and complications both intra and postoperatively were recorded and are presented in this study.

### Surgical Technique

Surgical resection of skin cancers occurred in an outpatient ambulatory care setting at a single institution in British Columbia, Canada. Keystone flaps were designed preoperatively by the PI according to Behan (2003), using either a keystone type I or keystone type III design, and outlined in a permanent marker. As previously described, if needed at the surgeon's discretion, adequate perforating blood vessels were identified using Doppler ultrasound. Anaesthesia was obtained using a combination of 1% lidocaine with epinephrine and bicarbonate and injected throughout the surgical field. The skin cancer was then excised with appropriate margins depending on suspected pathology and placed in a sterile container for histopathological analysis. Margins can vary depending on lesion size, location, biopsy report, and appearance. Margins routinely used for basal cell carcinoma (BCC) and squamous cell carcinoma (SCC) include clinical margins of >5mm and >7mm, respectively, and histopathological margins of at least a minimum of clear pathologic margins. If the margin for SCC is clear by less than 1 mm, a repeat procedure can be performed to ensure margins are clear to mitigate possible pathologist human error.

The edges of the keystone flap were then incised and elevated using blunt dissection down to fascia, with care taken not to undermine the delicate fasciocutaneous and musculocutaneous perforator blood vessels supplying the island flap. The flap was then advanced into the defect and secured using three 3-0 Monocryl anchoring sutures. The edges of the flap were advanced using bilateral V-Y advancement, secured with 3-0 Monocryl. Skin closure was done using 4-0 subcuticular Monocryl. The flap was cleaned with sterile gauze soaked in normal saline, Polysporin was applied, the patient was prescribed a 5-day course of antibiotics, given a list of postoperative wound care instructions, and had a follow-up appointment booked. Postoperative and follow-up pictures were acquired with patient consent and used for photographic evidence of wound healing and aesthetic outcome.

## Results

A total of 35 KDPIFs were performed on 32 patients by one surgeon, between February 2013 and February 2019 for the oncologic reconstruction of skin cancer defects isolated to the upper extremity. Of the 32 patients, 18 (56.25%) were male with an average age of 79.7 years (59-95 years), and females had an average age of 70.5 years (54-101 years) (Table 1).

**Table 1.** Patient Demographics.

Gender	N (%)	Average age (range)
Female	14 (43.8)	70.5 (54-101)
Male	18 (56.3)	79.7 (59-95)

**Figure 1.**
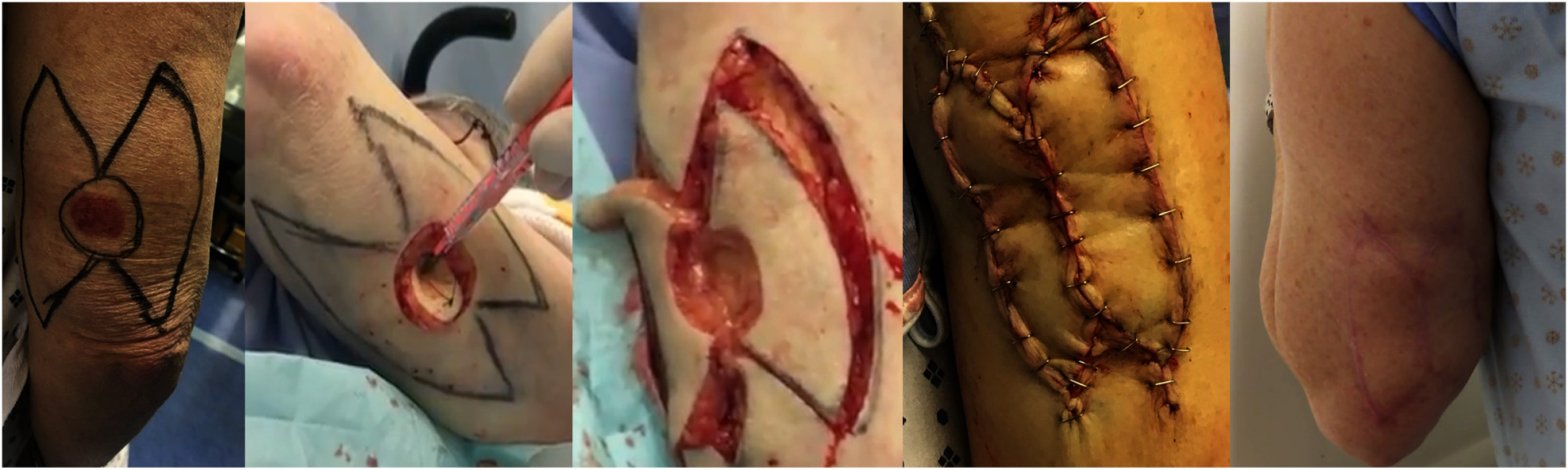
A 69-year-old female with a 2.8 × 2.6 × 1.0 cm squamous cell carcinoma (SCC) excised from her posterior arm. The lesion and the preoperative surgical double keystone markings are shown. Intraoperative lesion defect with keystone type III flap elevation is shown, followed by intraoperative approximation with staples. The patient is seen 9 months postoperative (far right). Patient was not included in the study due to timing of the study but is shown for educational purposes. The surgical technique described in this study was utilized for this patient.

**Figure 2.**
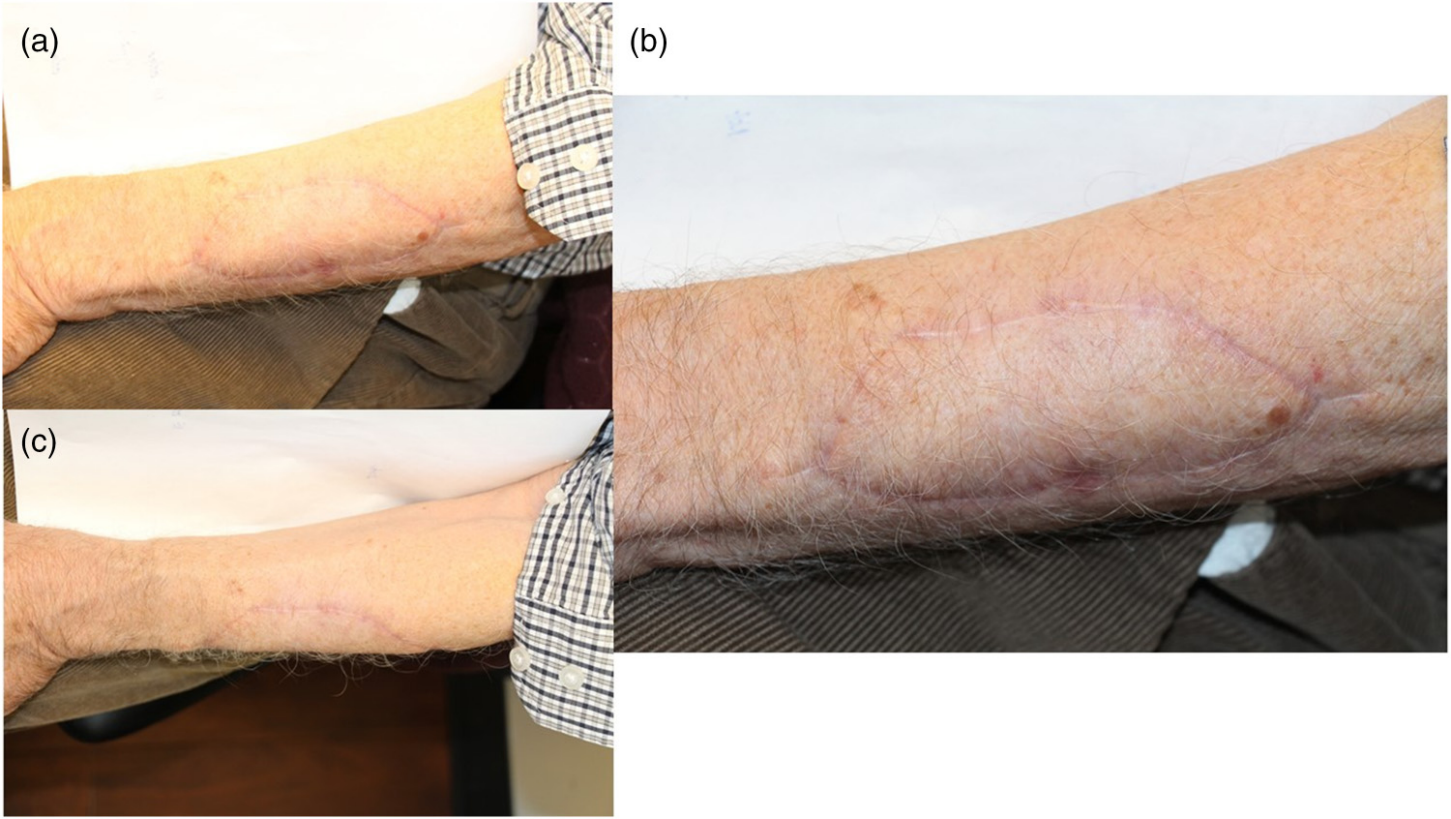
A 78-year-old male with a 5 × 4 cm basal cell carcinoma (BCC) on his left forearm (a, b, c). All photos were taken 5 months after surgical repair.

For all surgeries, 34 were keystone type I (97.14%) and 1 (2.86%) was keystone type III (“double keystone flap”, Figure 1). An ultrasound Doppler probe was used preoperatively to identify adequate perforator blood vessels in 11 of the 35 (31.4%) flaps. Histopathological reports revealed that of the 35 skin cancer excisions, the following diagnoses were identified: 14 (40.00%) BCCs (Figure 2), 11 (31.43%) SCCs, 9 (25.71%) melanoma skin cancers, and 1 (2.86%) actinic keratosis (Table 2).

**Table 2.** Etiology of Defects.

Lesion Pathology	Number of lesions (%)
Basal cell carcinoma (BCC)	14 (40.0)
Squamous cell carcinoma (SCC)	11 (31.4)
Melanoma	9 (25.7)
Actinic keratosis	1 (2.9)
All	35 (100.0)

**Figure 3.**
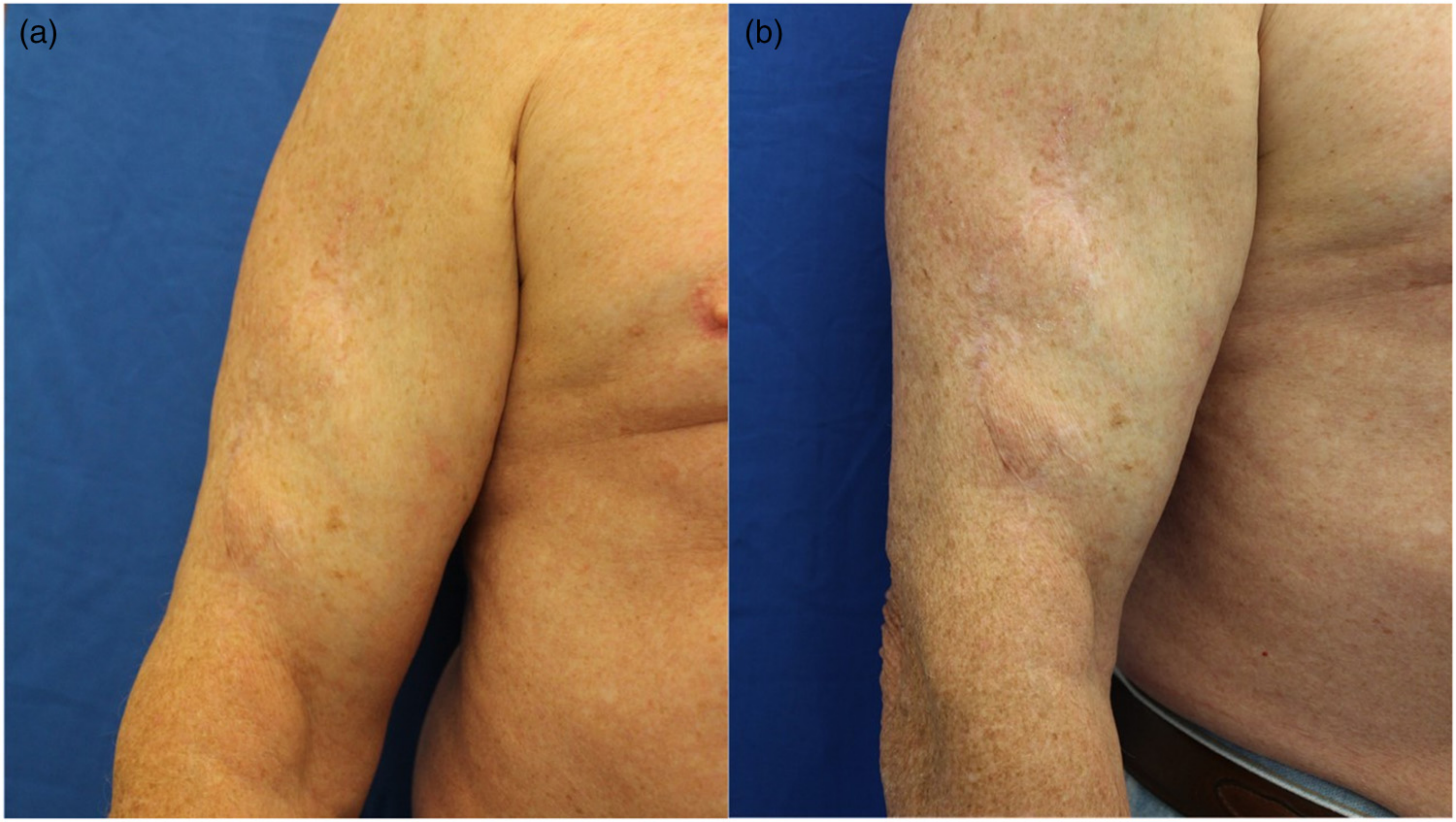
A 76-year-old male with a 2 × 1.5 × 0.7 cm melanoma. Two angles are shown (a, b) at 24 months postoperative. Note the absence of the contour deformity. Patient was not included in the study due to timing but is shown for educational purposes. The surgical technique used and described for patients in this study was performed for this patient.

The average area of defect excision was greatest and least among melanoma (12.3; 6.3-14.1) and actinic keratosis (4.24 cm^2^), respectively (Table 3). A melanoma reconstructed is shown in Figure 3.

**Table 3.** Average Defect Size of Excision Stratified by Lesion Pathology.

Lesion Pathology	Average area and range of defect excision (cm^2^ ± SD)	Range of defect excision (min–max cm^2^)
Basal cell carcinoma (BCC)	11.7 ± 8.34	3.5 to 31.4
Squamous cell carcinoma (SCC)	14.6 ± 8.59	5.7 to 31.4
Melanoma	12.3 ± 2.72	6.3 to 14.1
Actinic keratosis	4.24 ± 0.00	n/a
All	13.81 ± 7.33	3.5 to 31.4

The flaps were performed exclusively in the upper extremity with lesions removed and repaired on the arm (n = 14; 40.0%), hand (n = 8; 22.9%), elbow (n = 4; 11.4%), forearm (n = 4; 11.4%), shoulder (n = 3; 8.6%), and wrist (n = 2; 5.7%) (Table 4). The average area of defect excision for all lesions is 13.81 cm^2^ (3.5-31.4 cm^2^) with the forearm (15.9; 6.9-31.4) and wrist (5.4; 3.5-7.9) having the largest and smallest average elliptical areas, respectively. A forearm SCC defect repaired is shown in Supplementary Figure S1.

**Table 4.** Location of Skin Cancer Excision on Upper Extremity.

Location	Number of cases (%)	Average area of defect excision (cm^2^ ± SD)	Range of defect excision (cm^2^)
Arm	14 (40.0)	15.6 ± 6.86	3.5 to 10.2
Hand	8 (22.9)	6.9 ± 2.15	4.1 to 11.0
Elbow	4 (11.4)	14.1 ± 6.02	9.4 to 22.0
Forearm	4 (11.4)	15.9 ± 10.70	6.9 to 31.4
Shoulder	3 (8.6)	8.3 ± 4.51	5.9 to 14.1
Wrist	2 (5.7)	5.4 ± 3.12	3.5 to 7.9
All	35 (100.0)	13.81 ± 7.33	3.5 to 31.4

The largest defect reconstructed was 31.42 cm^2^. No intraoperative (partial or complete flap loss) or postoperative complications (eg wound dehiscence, infection, hematoma, and seroma) occurred during review of the procedure and postoperative charts. There were no cases of postoperative lymphedema or long-term sensory loss.

## Discussion

Skin defects as a result of oncologic resection require appropriate skin coverage. The mainstay for closure of skin excisions has been skin grafting, despite its donor site morbidity,^2^ relatively poor cosmesis^2^ and extended healing time.^3^ However, closure by direct or local flaps on the extremities is generally poor due to skin tension. As a result, the KDPIF, originally described by Behan in 2003, has been proposed as an effective local flap technique.^5^ In this study, we sought to determine the size of defects effectively repaired, the complications associated with the keystone flap procedure, and the overall aesthetic outcome of this procedure on skin defects located on the upper extremity.

There is limited literature on the keystone flap for lesions on the upper extremities compared to other anatomical locations.^15^ To the best of our knowledge, this study contains the highest number of excisions repaired with the keystone flap isolated to the upper extremity by a single surgeon and at a single institution. Given the varying lesion pathologies, locations on the upper extremity, size of skin defects, and absence of complications, it appears that the keystone flap is an effective skin closure technique for the upper extremity. This is in agreement with numerous studies that have concluded its advantages over primary closure and skin grafting, such as reduced tension, improved aesthetic outcome, absent dog ear resection, increased equipment simplicity, reduced splinting, and absent distant donor site defects.^5,16–19^ Furthermore, despite this study's hospital site not having any hand therapy available, none of the patients in this study attempted postoperative hand therapy and all regained full range of motion. Regarding prophylactic antibiotic usage, the PI considers the patients in this study as high risk for tissue compromise and infection and therefore was indicated for such. The majority were elderly with numerous medical comorbidities and as with other reconstructive techniques, flap dissection and tension at the site of closure impairs surrounding skin and subcutaneous tissue vascularity.

When comparing the keystone flap, an island flap, to free flaps or flaps with a single perforator performed on defects located on the lower extremity, decreased operative time given its relative simplicity and favourable cosmesis occurs.^20,21^ Although future studies should determine whether this trend continues with defects isolated to the upper extremity, given the keystone flap permits the transfer of identical local tissue with ideal tissue characteristics, it is feasible to predict that the benefits will remain true.

When designing the flap, there are a few pearls we believe are helpful for those considering incorporating the keystone into their own practice. First, when assessing closure of the V-Y advancement flaps on either end of the proposed skin island, it is possible to use the pinch test to assess whether there is sufficient laxity at the opposite ends to achieve closure. This may be extra beneficial when approaching defects at different levels of the upper extremity and thus varying amounts of laxity. However, in this study, the location of the defect did not affect flap design. Second, if there is concern regarding flap viability, such as in larger defects or if skin quality is poor, one may always increase the number of perforators and thus vascularity by increasing the flap size. Third, like the previous point, one can always use a Doppler and/or infrared thermography to identify the location and size of perforating vessels to include in the flap.

When concerned about incomplete excision margins, as with other reconstructive techniques, one may always consider a delayed approach such that the pathology and excisional margins results return prior to repair with the keystone flap. This will eliminate the risk of further subsequent repeat excision and repeat repair. However, if this approach is not taken and a patient has a positive lateral margin after reconstruction, the patient should receive surgery again. This subsequent surgery should excise the vertical incision line near the lateral margin with a repeat clinical margin and the keystone flap is readvanced. If there is concern regarding readvancement, a second keystone flap can be elevated to provide sufficient tissue for coverage of the defect. This would therefore change the reconstruction from a keystone type I to a type III.

In the case of melanomas, the study site did not have access to sentinel lymph node biopsy or general surgeons who were able to perform this procedure. Therefore, melanomas were not treated if stages were higher than T1A. As a result, the senior author took a stepwise approach to melanoma excision and reconstruction. First, the index biopsy, typically performed by the referring physician, is diagnostic. Only patients with lesions less than 1mm in thickness are seen by the PI. Thereafter, an excisional staging biopsy with 1 to 2 mm margins followed by closure with a purse string flap takes place. This ensures the maximal depth of the lesion remains to be less than 1 mm. Finally, excision with wide clinical margins of at least 1 cm with keystone flap reconstruction occurs. This ensures the melanoma has completely been removed.

Figure 1 shows a patient who underwent a type III design (“double keystone flap”) for a 2.8 × 2.6 × 1.0 cm SCC excised from her posterior arm. Double keystone flaps permit the closure of larger skin defects than single keystone flaps. In addition, it enables the transposition of two islanded flaps, which may be necessary for areas of high skin tension, such as when readvancement is required for new defects that initially had positive excisional margins. It is also important to note that no contour deformity occurred, despite a 1 cm thick skin excision. Although keystone flaps are regularly used and described for head, neck, trunk, and extremity skin excision reconstructions, a case report by Yunir et al has demonstrated the success of the double keystone for a large skin excision defect located at the suprapubic area and scrotum.^22^ The ability for double keystone flaps further demonstrates its versatility among various anatomical locations and size defects where a single keystone flap would not be suffice for excision closure.

A study of 60 keystone flaps, with five cases of upper extremity traumatic defect reconstructions on the upper extremity, reported two keystone flaps with wound healing complications (separation and epidermolysis) with no surgical site infections.^23^ Despite only five upper extremity cases, wound healing complications were more prevalent in reconstructions on the lower than the upper extremity, supporting its comparable effectiveness for reconstructions on the upper limb. Future studies with a greater number of cases should directly compare the complication rate of upper versus lower extremity keystone flaps. We recommend that to decrease the likelihood of separation and epidermolysis, if the incisions rub against the patient's clothing, mechanical forces may be decreased by covering the wound with a dry dressing. And if the flap is over a joint, it would be helpful to immobilize the joint in the position of function for approximately 10 days to avoid unwanted forces.

The average excision size of lesions when the Doppler is used versus not in this study is 15.52 cm^2^ and 11.53 cm^2^, respectively. This is a modification to the original surgical technique described by Behan, where no Doppler ultrasound was used preoperatively. Doppler usage to identify perforating vessels has been reported previously and is useful to confirm myocutaneous or fasciocutaneous vascular perforators with flaps that are relatively larger. A study by Patel et al describes the usage of smartphone thermal cameras to locate perforators when intraoperative Doppler angiography fails.^24^ Thermography with smartphone thermal cameras has shown high concordance with CT angiography and improved ability to identify perforators in the lower extremity over Doppler angiography.^25^ Although Doppler was adequately effective for patients in our study, other options for identifying perforators for more complex reconstruction on the upper limb should be studied.

### Limitations of the Study

A limitation of this study is that other reconstructive techniques were not directly compared to the keystone flap. In addition, it would be beneficial to determine the aesthetic outcomes of the keystone flaps with standardized aesthetic and patient satisfaction assessments to further elucidate the keystone flap's effectiveness in restoring form and function. Despite these limitations, the absence of any complication and good cosmesis in our study supports the effectiveness of keystone flaps for skin excision defects on the upper extremity.

## Conclusion

Given the absence of any perioperative complication, the varying histopathological diagnoses and size of skin cancers successfully reconstructed on all locations of the upper extremity, and good cosmesis, the keystone flap is a successful skin closure procedure for skin cancer excisions up to 31.42 cm^2^ of any location or pathology on the upper extremity.

## Supplemental Material

sj-tif-1-psg-10.1177_22925503221094106 - Supplemental material for Keystone Flap for Closure of Skin Cancer Defects on the Upper Extremity Supplemental material, sj-tif-1-psg-10.1177_22925503221094106 for Keystone Flap for Closure of Skin Cancer Defects on the Upper Extremity by Travis Gordon, Andrew P. Golin and Alexander Anzarut in Plastic Surgery
