# Supplementary figures and images for: Keystone Flap for Closure of Skin Cancer Defects on the Upper Extremity
Source: Plast Surg (Oakv). 2022 Apr 21;32(1):47–53. doi: 10.1177/22925503221094106 (PMC10902476; doi:10.1177/22925503221094106)

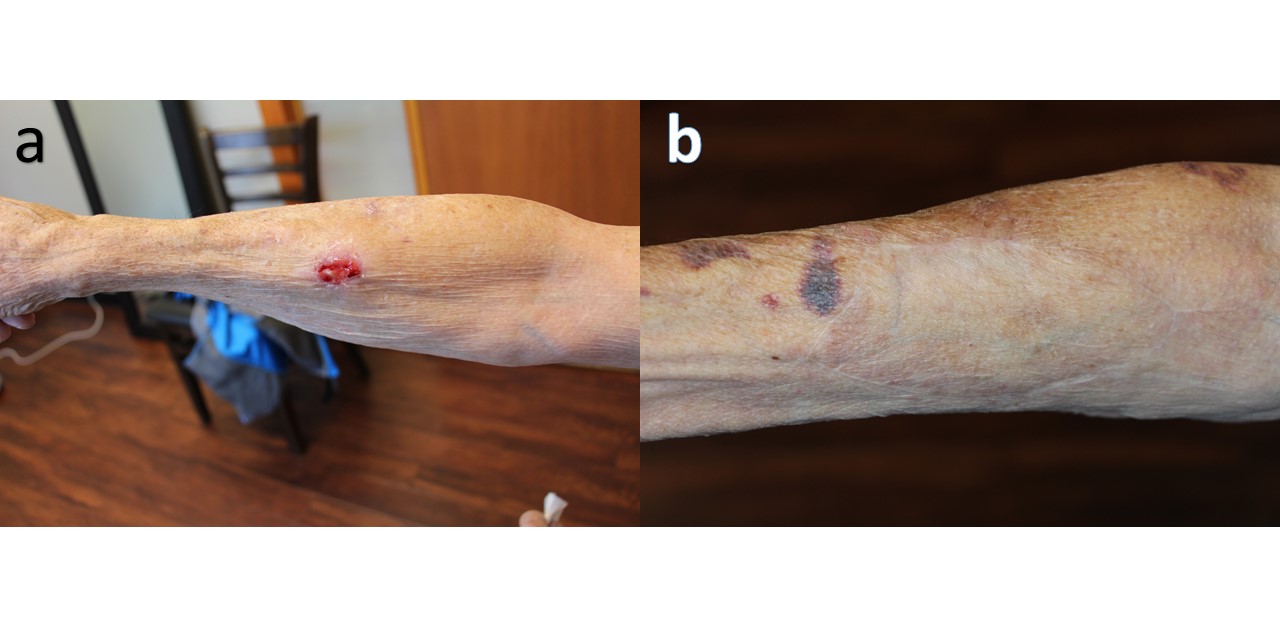

Supplement: sj-tif-1-psg-10.1177_22925503221094106 - Supplemental material for Keystone Flap for Closure of Skin Cancer Defects on the Upper Extremity [file sj-tif-1-psg-10.1177_22925503221094106.tif]
